# Considerations about the Signal Level Measurement in Wireless Sensor Networks for Node Position Estimation

**DOI:** 10.3390/s19194179

**Published:** 2019-09-26

**Authors:** Stelian Dolha, Paul Negirla, Florin Alexa, Ioan Silea

**Affiliations:** 1 Department of Automation and Applied Informatics, Politehnica University Timişoara, Timşoara 300006, Romania; stelian.dolha@gmail.com; 2 Department of Communications, Politehnica University Timişoara, Timişoara 300006, Romania; florin.alexa@upt.ro

**Keywords:** sensor networks, RSSI (Received Signal Strength Indicator) acquisition, adjusted transmission power, sensor location estimation, non-linearity of the measured values

## Abstract

Wireless Sensor Networks (WSN) are widely used in different monitoring systems. Given the distributed nature of WSN, a constantly increasing number of research studies are concentrated on some important aspects: maximizing network autonomy, node localization, and data access security. The node localization and distance estimation algorithms have, as their starting points, different information provided by the nodes. The level of signal strength is often such a starting point. A system for Received Signal Strength Indicator (RSSI) acquisition has been designed, implemented, and tested. In this paper, experiments in different operating environments have been conducted to show the variation of Received Signal Strength Indicator (RSSI) metric related to distance and geometrical orientation of the nodes and environment, both indoor and outdoor. Energy aware data transmission algorithms adjust the power consumed by the nodes according to the relative distance between the nodes. Experiments have been conducted to measure the current consumed by the node depending on the adjusted transmission power. In order to use the RSSI values as input for distance or location detection algorithms, the RSSI values can’t be used without intermediate processing steps to mitigate with the non-linearity of the measured values. The results of the measurements confirmed that the RSSI level varies with distance, geometrical orientation of the sensors, and environment characteristics.

## 1. Introduction

In order to be able to easily go through the work, it was considered necessary, from the beginning, to present the synthesis of the notations and abbreviations used. The notations and abbreviations, in the order in which they appear in the paper are presented in Table 1.

Received Signal Strength Indicator (RSSI) represents a measure of the signal power on a radio link. The link can be affected by several channel parameters also known as channel conditions causing variations of RSSI level, such as distance between nodes, radio transmission medium (e.g., air, water), physical obstacles, geometrical orientation of the nodes and interference with other radio transmission equipment and reflected radio waves. Given this sensitivity to various parameters, RSSI is widely chosen for estimating distance, node localization, motion detection [1], tracking algorithms and security mechanisms (e.g., intruder detection) as in [2]. 

Wireless sensor networks slowly move from academic and industrial environments to people in hospitals, smart energy grids, urban monitoring, sustainable development [3] and finally in smart homes as Internet of Things devices [4]. These environments are prone to different interferences and each ambience factor needs to be handled separately. Moreover, the impact of wireless medium [5,6], temperature and humidity [7] on RSSI need to be taken into consideration on WSN with greater distance between nodes. Finally, the monitoring of QoS [8] in these processes became an essential part of RSSI and ToA based solutions. Together with the above environment considerations, these factors have established reliable and well-founded WSN localization methods in both two dimensional as well as three dimensional [9] systems.

Despite the lack of precision, RSSI is widely used today in most of WSN localization algorithms [10]. A comprehensive analysis of basic principles and techniques used in the localization algorithms, categories of these algorithms and localization schemes has been covered in [11,12]. Although these methods are affected by the subject of this this paper, they have not been benchmarked or detailed throughout our study and make part of further research. 

Novel calibration schemes and filtering solutions developed in the last years. A new method described in [13] focuses on WSN localization for long-range wireless sources using low computation complexity and reaches the high accuracy under Gaussian noise. Also, in [14] an algorithm with high localization accuracy when the signal to noise ratio is high is proposed which can improve the accuracy without the need of modifying the underlying hardware. Previous proposed models based on Bayesian or Particle filter noise reduction solutions [15] are still computationally expensive and are not always fit for convoluted indoor environments [16,17]. 

The first problem in an application with sensor nodes is locating or estimating their position. The basic concepts of location estimation start from the assumption that there is a central node, also called base station and additional fixed or mobile nodes communicating with the base station and taking measurements of different parameters like RSSI, angle of arrival (AoA), time of arrival (ToA) and time difference of arrival (TDoA) as described in [18]. A comparative analysis of these parameters is documented in [19].

In ideal circumstances, also called free space model, the relationship between signal attenuation and distance can be expressed as (1), according to [20], which describes with limited accuracy the path loss, based only on distance and frequency. 

PL(d_0_) = −32.44 − 20log(f_c_) − 20log(d_0_), <dB> (1)

PL is the path loss, f_c_—the central frequency and d_0_—the distance between nodes. Besides RSSI, the other measurements are more difficult to be performed as they require specialized hardware components usually not found of wireless sensors due to design to cost constraints and miniaturization efforts. RSSI measurement mechanisms are nowadays common built-in features of radio transceiver chips making RSSI an affordable technical solution. Although, RSSI measurements are very sensitive to environmental changes and often require intensive calibration processes, both offline and online, as described in [21]. 

A second problem in wireless networks is the estimation of the distance between the nodes in order to increase the energy-efficiency of the network. Energy-efficient wireless sensor networks are characterized by increased autonomy given by low power consumption and auxiliary energy harvesting mechanisms. In a traditional system, a wireless sensor is powered from batteries or accumulators as main power supply. Each wireless sensor includes the following four main blocks: radio transceiver, processing unit (usually a microcontroller), signal acquisition and filtering circuitry and an output stage (for local process control). 

In order to minimize the power consumed by the microcontroller, different techniques can be applied, usually activating sleep or power down modes, reducing the operating frequency and shutting down unused peripherals. Similar power down modes can be activated for the radio transceiver which can also adjust the transmission power, therefore, reducing the current consumption.

**Figure 1:**
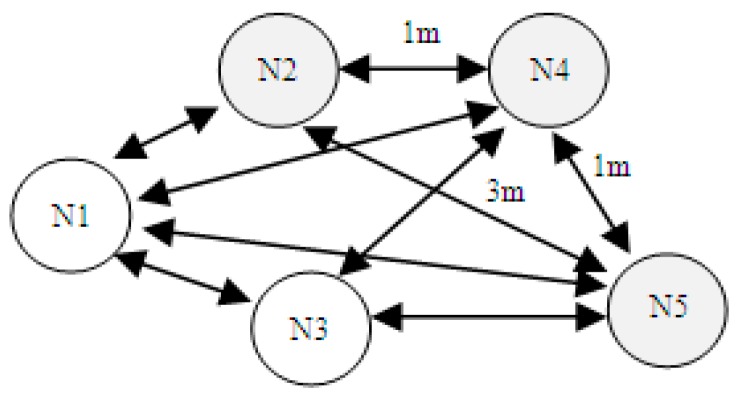
Example of a wireless sensors network with different distances.

In Figure 1, an example of a small wireless sensors network with different distances between the nodes is being shown. Supposing an interpolation table, showing necessary power to transmit a packet over a defined distance is built upon RSSI information and available at runtime, the following power reduction scenarios are possible:

Node N2 transmits a packet to node N5 with a specific power level (L5), being able to reach node N5 located 3 m away. 

Node N2 transmits a packet to node N4 with a specific power level (L4), where L4 < L5, being able to reach node N4 located only 1 meter away. In this way, dynamic adjustment of transmission power is possible.

Moreover, if the whole network autonomy is critical, single-hop transmissions requiring increased power consumption can be replaced by multi-hop transmissions based on a network autonomy maximizing algorithm. 

However, wireless node network systems are scalable between certain limits, imposed on the one hand by the address space (the number of bytes available), and on the other hand by the algorithms for detecting and locating the nodes in order to restore the network, when a node disappears (signal from it is too small).

The paper shows that using a simple setup you can perform measurements that validate (node by node) a wireless sensor network architecture. The authors consider such an operation obligatory, by which it is allowed, in fact, to test the hardware scalability and implicitly the stability of the software algorithms, that is the limits of good functioning. The low level of the RSSI value can be interpreted as the disappearance of a node in the network, and the software algorithms used become more complex. Through the research carried out, the authors recommend carrying out signal measurements, on the spot, when considering the extension of the sensor network area or the scaling of the number of sensors. Aspects of the signal level measurements (using RSSI) are presented in the paper when the supply voltage of the nodes and the orientation of the sensors (at the same location) do not always remain the same.

## 2. Materials and Methods

The results and experiments presented in the paper are useful for carrying out at least two applications:

(i) A system for identifying the position of vacant places in a parking lot, when the occupancy rate exceeds 75% and the vacant places are difficult to identify;
(ii) A system for reading energy consumption from each of the apartments on the scale of a multi-level building. The architecture of both applications is based on a major node and several fixed or mobile secondary nodes. Between the nodes, in different variants, links are established. The link can be affected by several channel parameters also known as channel conditions causing variations of RSSI level.

In this paper, a system for RSSI acquisition has been designed, implemented and tested in order to use this metric as an input for future development of distance and location estimation algorithms. The goal of the system is to allow the user to measure the RSSI levels between active nodes in the system and in both directions. Both directions mean that it is not only interesting to measure the RSSI level for a transmission between Node N2 and node N4 (of Figure 1), but also for a transmission between node N4 and node N2. This aspect was taken into consideration for situations in which the two measurements would be different because the parameters of the wireless sensors are different. Two examples of such parameters are the transmission power level and the power supply voltage.

Considering the system in Figure 1, N1 is the central node and N2, N3, N4, and N5 are remote nodes. The system needs to be able to measure the distance from the central node to each remote node but also between any other 2 remote nodes. The difference between central and remote nodes is that the central node has a UART (Universal Asynchronous Receiver/Transmitter) connection to another device, in this case, a PC (Personal Computer). This wired communication link is used to trigger commands and collect data from the wireless network by the help of a dedicated desktop user interface.

Apart from the level of the emission power and the level of the supply voltage, the direction in which the signal is sent/received greatly influences the quality of the communication. The experiments presented in the paper highlight its importance, especially for the applications based on mobile nodes.

### 2.1. System Description

**Figure 2:**
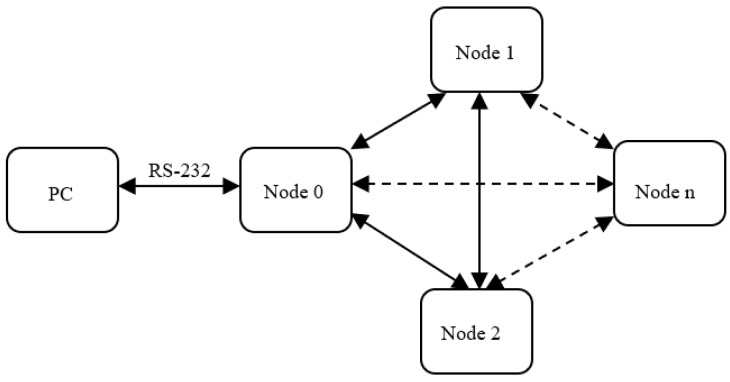
Received Signal Strength Indicator (RSSI) measurement system architecture. Although physically distributed, the access to network data is done via a central node, Node 0.

Figure 2 describes the measurement system architecture. The system has n nodes denoted Node 0, …, Node n, and Node 0 communicates with a PC through an RS232 interface. Nodes N1, N2, …, Nn, are mobile nodes that will can be located at different points in the area/building where the experiments are performed. Our experimental setup used the main node (N0) and successively one mobile node to perform the measurements. The mobile node is placed in the position and with the desired orientation. We have thus proceeded to have results for the same node in different positions/locations; the behavior of a node was studied. Of course, by using all the nodes you can make determinations on the entire sensor network, in architecture from a particular case.

**Figure 3:**
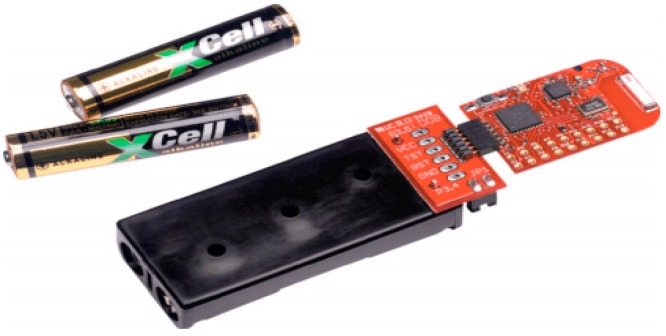
eZ430-RF2500T wireless node.

The wireless sensors used in the system are eZ430-RF2500T from Texas Instruments. They are built around a low power microcontroller, the MSP430F2274 derivate, from Texas Instruments and a low power 2.4 Ghz transceiver, the CC2500 from Chipcon. The sensor is powered by a 3 V battery pack (2 × 1.5 V AAA batteries). The wireless node is shown is Figure 3.

The node has an onboard radio antenna, LEDs, digital input-output pins and a programming/debugging interface, providing extension possibilities. The operating CPU frequency of the microcontroller is 1 Mhz. The CC2500 is a 2400–2483.5 Mhz transceiver operating in ISM band, with a programmable data rate between 1.2–500 kBaud, programmable transmission power level, and digital RSSI and LQI (Link Quality Indication) outputs as described in [21].

### 2.2. Communication Protocol

Two similar communication protocols have been implemented. One is handling the wired communication on RS232 interface between the PC and the central node and the other is handling the wireless communication between the wireless nodes.

The protocols are based on the following telegrams.

(a) Control telegram
(b) Answer telegram

**Figure 4:**
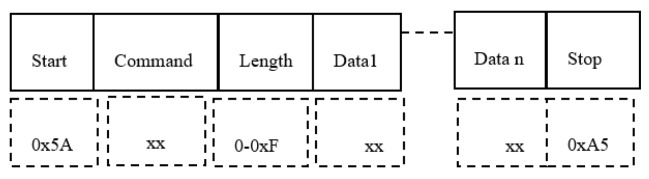
The structure of a control telegram.

Figure 4 describes the structure of the control telegram. 

We observe the Start byte, then the Command byte, followed by the Data field length (bytes), the Data bytes (Data 0, …, Date n) and the Stop byte. 

**Figure 5:**
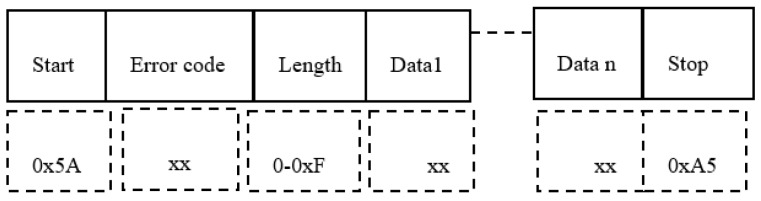
The structure of an answer telegram.

Subsequently, Figure 5 describes the structure of the answer telegram.

The answer telegram is delimited by a Start and Stop indicator. The *Error code* is encoded on one byte, followed by the length indicating the number of data bytes received. Data is being sent MSB first and only in case of a successful command execution. The same format of the answer telegram is preserved for both the wired and wireless protocol. Table 2 shows the supported control commands.

The software allows scalability of the control services supported by each node allowing the user to adjust the number of services depending on the hardware capabilities in terms of RAM and ROM memories footprints available. 

**Figure 6:**
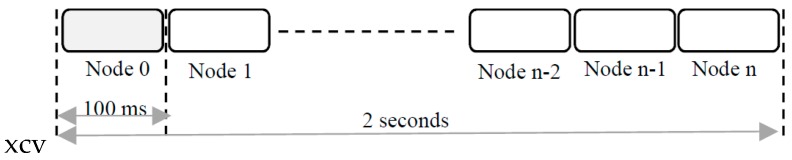
TDMA mechanism used for network scan.

A communication is always triggered by the desktop application. The central node executes the command locally if the central node is the destination or forwards the command to other remote nodes from the network. At a specific time, only one node is allowed to send data so that channel interference is being avoided. An exception to this rule was allowed for implementation of command NET_SCAN (Network scan) which polls the network for finding available nodes. In order to avoid interference, a time division multiple access (TDMA) mechanism was implemented as shown in Figure 6. The central node scans for 2 s the network and retrieves the total number of nodes found and their associated ID numbers. For each node, an 8 bit identification number (ID) is being assigned. The ID is programmed in the flash memory and can’t be overwritten during runtime.

The central node sends a broadcast message in time slot 0. Each node receives the message and sends back the answer in its own time slot of 100 milliseconds.

### 2.3. Communication Protocol Parameters

The wired communication uses RS-232 interface and UART protocol. The UART frame setting is 8N1, meaning 8 data bits, LSB first, no parity and 1 stop bit. 

The wireless communication is set to a frequency of 2400 MHz and a 250 kBaud rate. The maximum number of data bytes in a transmission is set to 16. The limitation is necessary due to low RAM memory constraints of MSP430F2274. The software allows adjustment of the data transmission and reception buffers providing easy porting to other hardware architectures.

### 2.4. RSSI Indication

**Figure 7:**
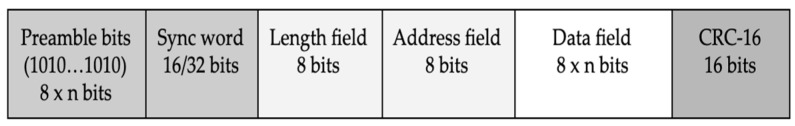
CC2500 radio transmission packet structure.

When a package is received, the data is stored in internal RAM memory of the transceiver and can be retrieved for further usage via SPI interface. CC2500 can be configured to append the calculated RSSI to the received packet buffer. The value is stored in 2′s complement. Further processing of this value is necessary as there is a baud rate-dependent offset which needs to be subtracted. For a transmission rate of 250 kBaud, an offset of 72 dBm needs to be subtracted from the raw RSSI value, in accordance with [22]. In Figure 7, the structure of a packet is being described.

There are 4 preamble bytes and 4 sync bytes configured for the transmission. The communication protocol adds an overhead of 4 bytes to each packet being transmitted. Hence, the transmission duration (D) of a single data frame, carrying n bytes of data, at a transmission rate of 250 kBaud, can be calculated using (2).

D(s) = [16 × 8(fixed bits) + n × 8(data bits)] × 4 × 10^−6^, <s> (2)

## 3. Results

Starting from the idea that the radiation diagram of a sensor node is not isotropic, the paper shows the results of the RSSI factor measurements in several situations of geometric arrangement: inside and outside, in real environment. The interdependence between the voltage levels of the power supply, the non-linear variation of the transmission power level depending on the register settings, was determined for the sensors mentioned above.

Because the experiments were not performed under laboratory conditions and the environmental factors (temperature, humidity, pressure …) cannot be controlled/modified, the results reflect the situation of the measurements only due to the distance and geometric orientation of the sensor nodes in the real environment.

### 3.1. Current Consumption

The total current consumption is given by (3).

C_Total_ (mA) = C_CPU_ + C_Outputs_ + C_Transceiver_, <mA> (3)

C_CPU_ is the current consumed by the CPU and peripherals and it is defined usually by the operating speed, external circuitry and activated (powered) peripherals. C_Outputs_ is the current consumed by the outputs controlled by the CPU. In this case, there is only one output, a LED used as a package received visual indicator, toggling each time a new package was successfully received. Finally, C_Transceiver_ is the current consumed by the on-board radio transceiver which can be influenced by the operating mode of the transceiver: power-down, idle, RX active or TX active on one hand and by the level of transmission power if the operating mode in TX active.

The measure of interest is the total current consumption of the node when in TX active mode, as this would be the variable which could be modified dynamically by an energy-efficient data transmission algorithm. 

The communication protocol offers two control services, which can be used to measure consumption of the current during a transmission.

**Figure 8:**
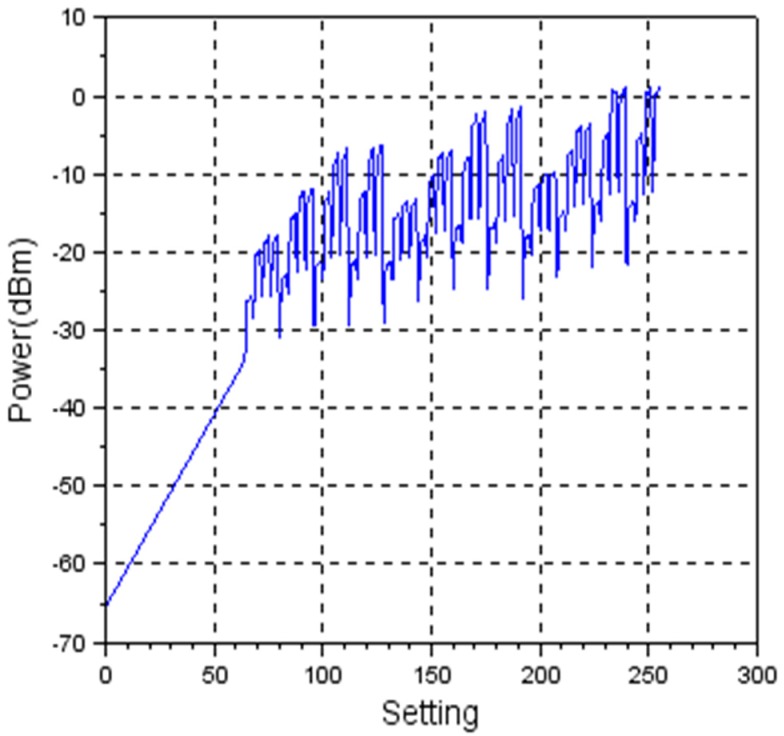
Non-linear variation of transmission power level vs. register setting.

Using TX_LEVEL command, the user is able to control the TX power level with a granularity of 192 power level steps. CC2500 provides a user writeable 8-bit control register for setting the TX power level. Figure 8 shows the dependency between the register value and TX power level.

**Figure 9:**
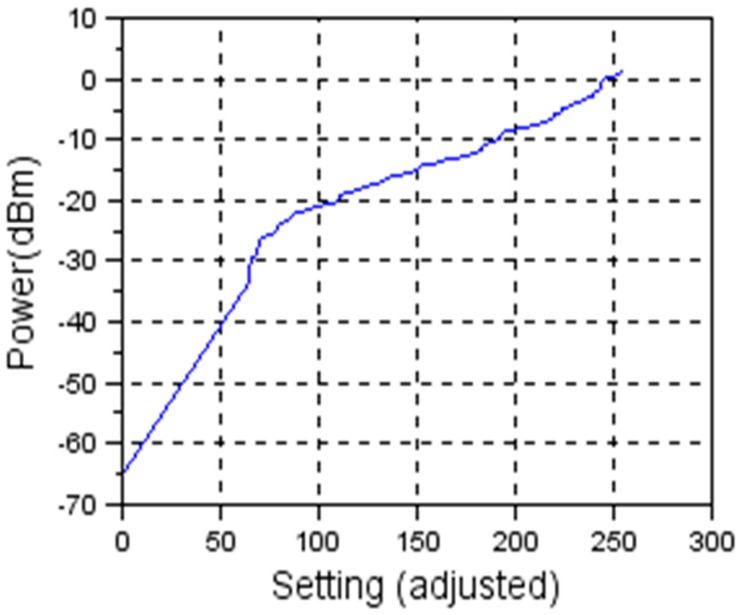
Linearization of transmission power level vs. register setting.

As the characteristic is not linear, the solution was to implement a look-up table in software, so that the non-linear characteristic is not transparent to the end user. In Figure 9 the obtained linear characteristic is being shown.

Using TX_STRESS command, a set of predefined data frames are being sent by a remote node while measuring the current consumption of the node during transmission. The current measuring device used in these experiments is Agilent 34401a multimeter. The device is able to take measurements at a rate of 200 samples/second, resulting in a sampling period of 5 milliseconds. 

The duration of a single 10 bytes transmission, at a rate of 250 kBaud, calculated using (2) is equal to 832 microseconds. As the duration of a single frame transmission is less than the sampling period of the measurement device, it is necessary to force the CC2500 to continuously transmit data frames to increase the measurement time window. Hence, the device will stay in TX active state for a user-defined number of packet transmissions. Two sets of current measurements were conducted in TX mode for all 192 different power level settings. For each measurement, 1000 packets were sent. 

**Figure 10:**
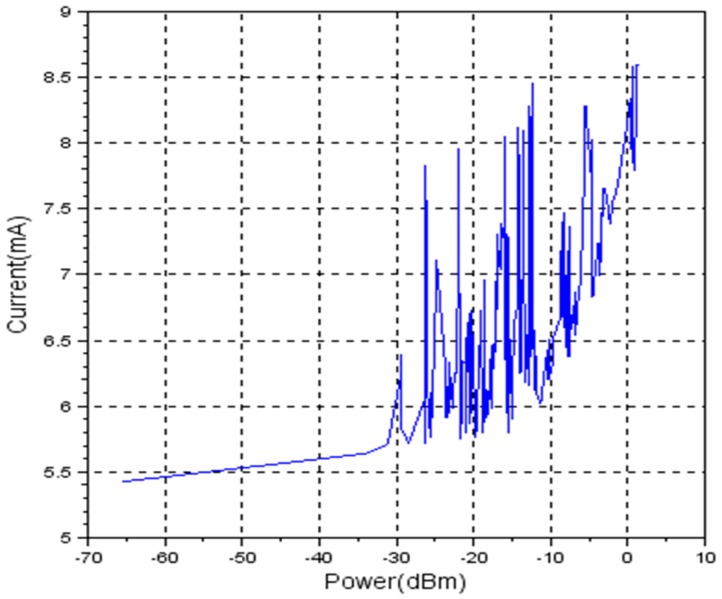
Current vs. transmission power for fully charged batteries (VBAT = 3.16 Volts).

For the first set, the wireless node was powered by two 1.5 Volts AAA batteries, fully charged. In Figure 10, the measurement results are being shown.

The graphic shows a non-linear characteristic.

**Figure 11:**
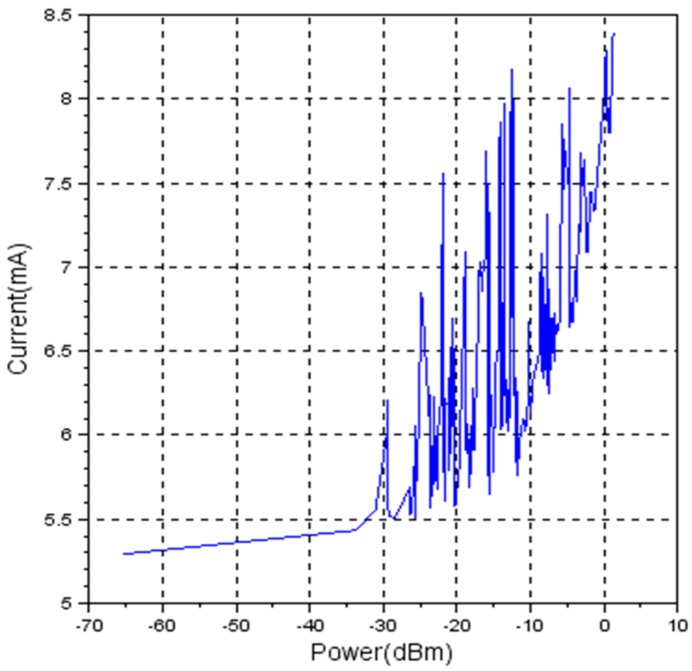
Current vs. transmission power for discharged batteries (Vbat = 2.768 Volts).

For the second set, the wireless node was powered by two discharged 1.5 Volts AAA batteries. In Figure 11, the measurement results are being shown.

The graphic in Figure 11 shows again a non-linear characteristic.

**Figure 12:**
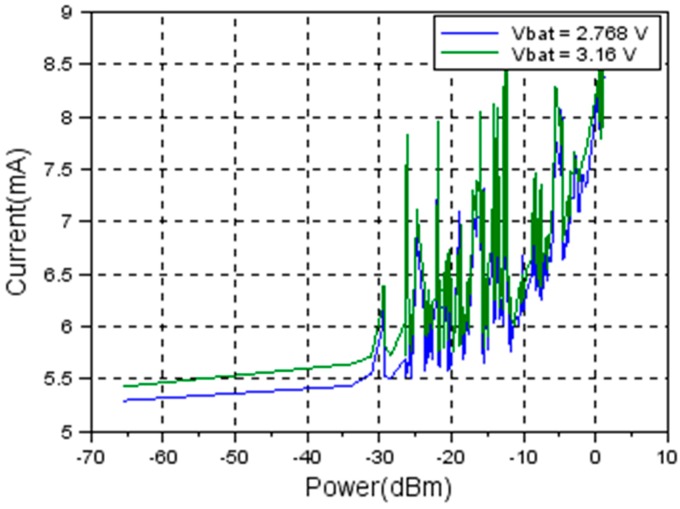
Current vs. transmission power. Comparison between fully charged batteries and discharged batteries.

By overlapping Figure 10 and Figure 11, the current consumption difference can be observed in Figure 12. As expected, the current consumption in case of the discharged batteries is lower. The non-linear characteristics are confirmed in [22].

For a sensor, the measurements of current made according to the emission power, with the sensor’s battery power being charged and discharged, differ. Therefore, the autonomy of such a sensor, estimated by measuring the battery voltage, influences the accuracy of the distance estimation, based on the RSSI measurement.

**Figure 13:**
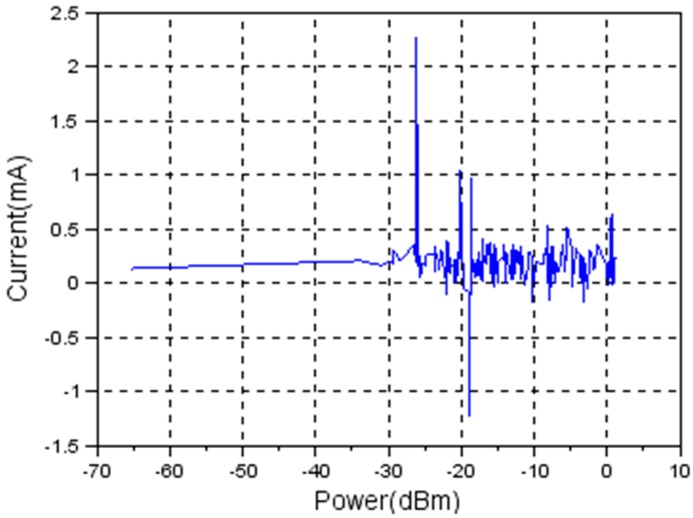
Current vs. transmission power. Current consumption difference caused by a voltage drop of 0.48 Volts.

Figure 13 shows the difference in current consumption when there is drop of 0.48 Volts in the power supply.

### 3.2. RSSI Measurements

**Figure 14:**
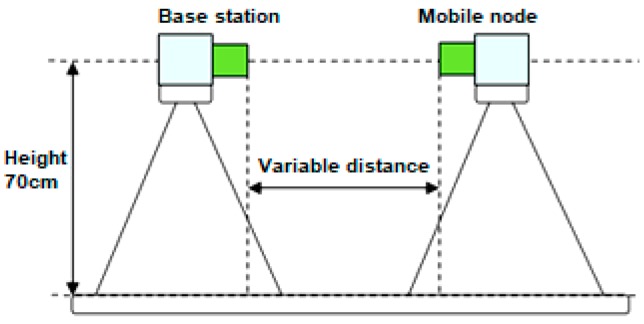
Indoor/outdoor measurement setup.

Multiple measurement sets were performed for evaluating the RSSI level in different conditions. The measurements were triggered using two control commands of the wireless protocol. First, a NET_SCAN command was sent to scan for active nodes in the action range. After having the list of modes available, GET_RSSI command was used to retrieve the RSSI level. GET_RSSI command works in two modes: if the source node is equal to central node, the RSSI levels between central node and a remote node are being measured. This process is called direct RSSI measurement. Indirect RSSI measurement is performed between any two nodes different than the central node. Using the two modes, any RSSI level between any nodes in the network can be measured. For outdoor and indoor measurements, the node positioning system described in Figure 14 was used.

#### 3.2.1. Outdoor Measurements

Using the system setup described in Figure 14, the first set of measurements was carried out in an open field covered by a concrete surface (parking lot). On a 31 m circle radius, no obstacles were standing in the line of sight, thus it was considered an environment with minimum interference probability.

**Figure 15:**
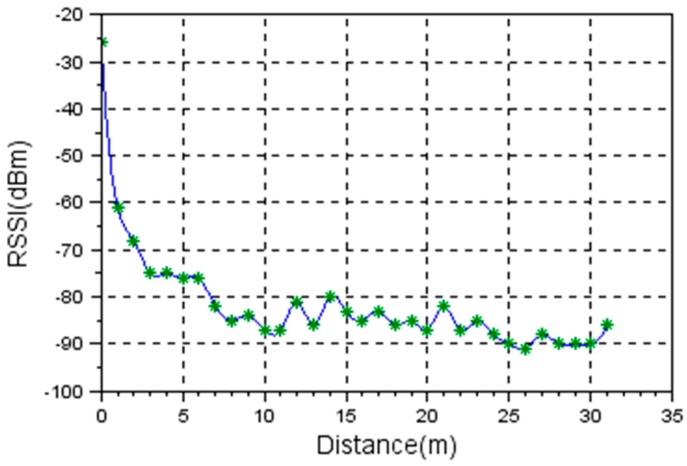
RSSI level vs. Distance. Open field measurements.

The base station position was stable during the measurement and the remote station was moved in 1 m steps away from the base station. Theoretically, in this setup, the only disturbance should be the reflection of the radio wave from the concrete ground (surface). In Figure 15, the results of the measurements are presented.

It is visible that in an open field, for a distance up to 10 m, the characteristic is linear, but the measurements degrade as the distance increases. The measurements were possible for a distance up to 31 m with maximum transmission power level set. Further away, the signal was too weak and the packets were lost. The range can be extended by usage of external antenna and proper antenna adaption circuitry.

#### 3.2.2. Indoor Measurements

**Figure 16:**
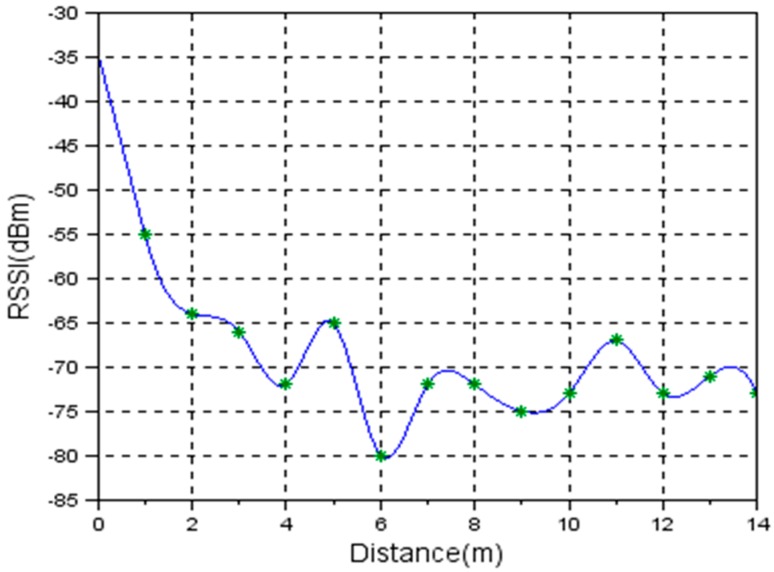
RSSI level vs. Distance. Indoor measurements on a 14 m long and 1.5 m wide corridor.

Using the system setup described in Figure 14, the first set of measurements was carried out inside a corridor, 14 m long and 1.5 m wide. The measurement assembly was centered on the longitudinal axis. The remote station was moved in 1 m steps away from the base station. Theoretically, in this setup, the measurements are affected by the wave reflections from the floor, ceiling and surrounding walls. In Figure 16, the results of the indoor measurements are presented.

Comparing Figure 15 and Figure 16, it is visible that for the same distance the RSSI level in an indoor environment is higher. The assumption is that due to multiple radio wave reflection paths, the signal power loss is reduced.

#### 3.2.3. Indoor Horizontal Orientation Measurements

For conducting the geometrical orientation measurements, the system in Figure 14 was used, with a fixed distance of 1 m between the base station and remote station. These experiments indicate if there is a change in RSSI levels in case of different positioning of the remote station relative to the base station. 

**Figure 17:**
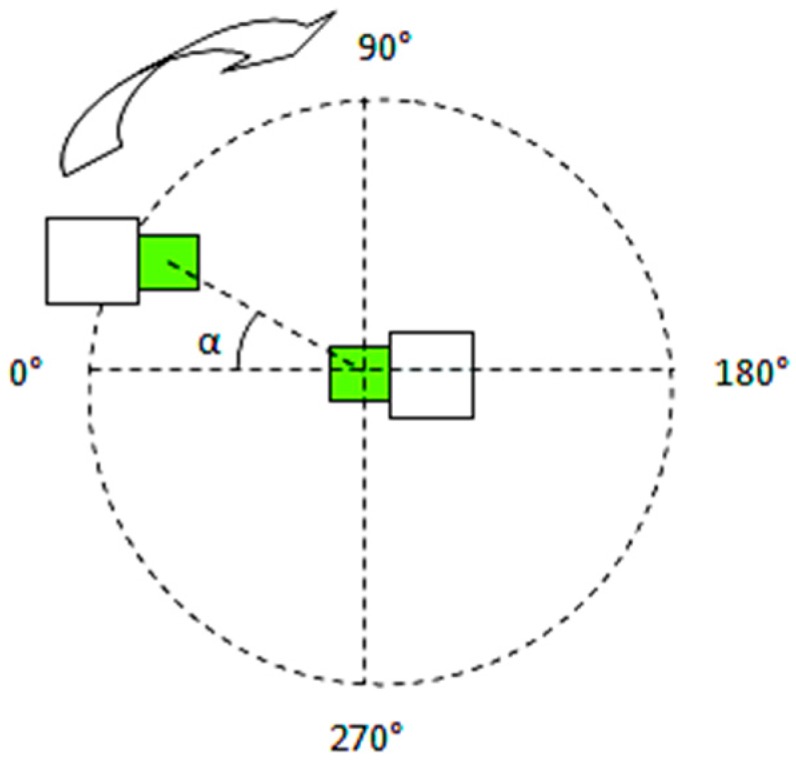
A 360° face-to-face measurement setup.

**Figure 18:**
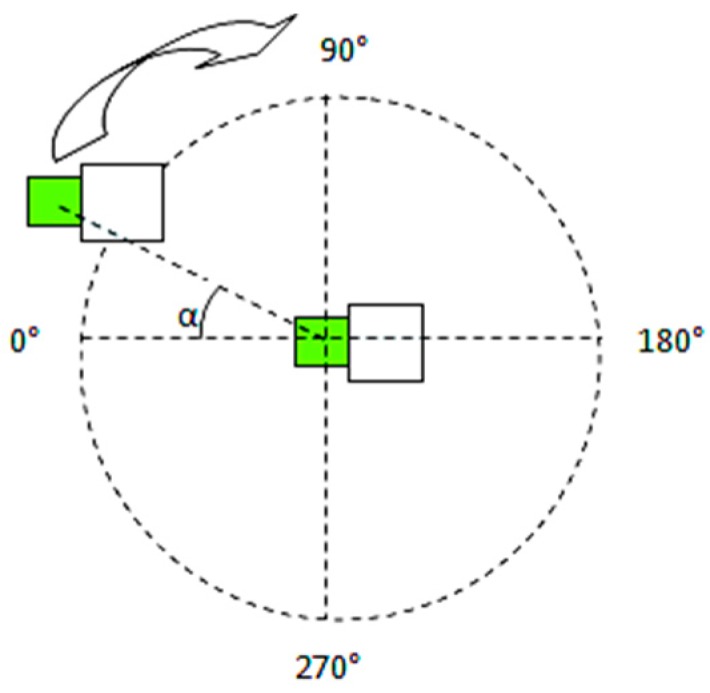
A 360° back-to-back measurement setup.

Two sets of measurements were done, one with the nodes facing each other and one with the nodes in a back to back positioning. A set of eleven measurements were done to cover a 360 degrees area around the base station, with a granularity of α = 30 degrees as shown in Figure 17 and Figure 18.

**Figure 19:**
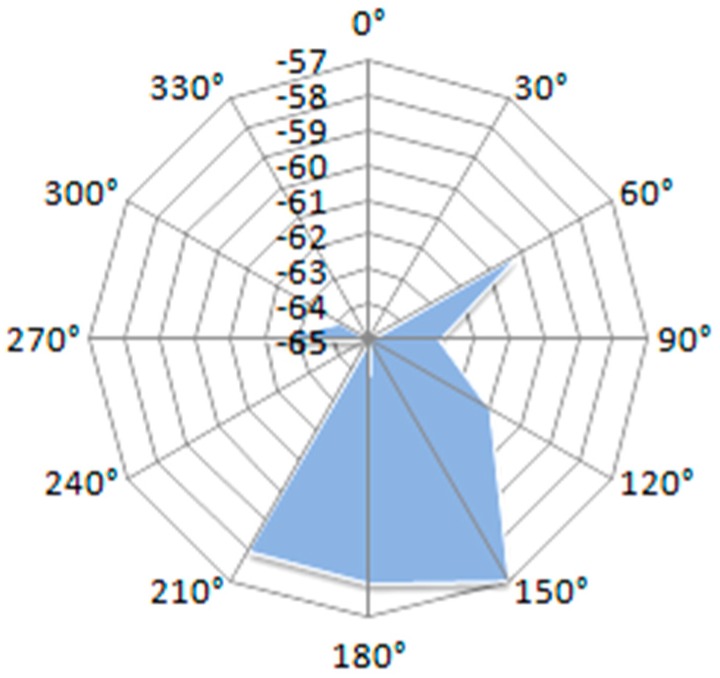
RSSI level vs. node orientation in a face-to-face measurement setup.

Figure 19 displays the results of the measurements conducted using the face-to-face measurement setup. Distance between nodes is 1 meter.

By keeping the base node fixed and rotating the mobile node, from the measurement chart it can be seen that the RSSI level differs considerably. The measurements were made every 30 degrees.

**Figure 20:**
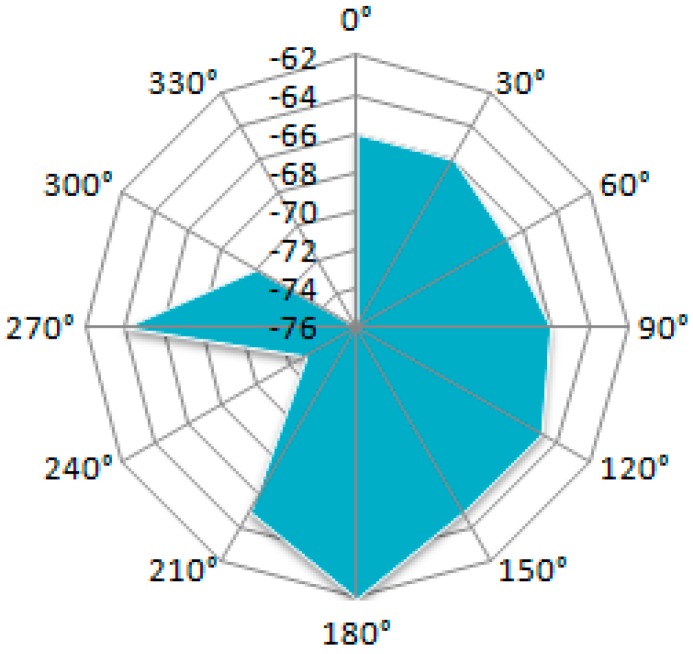
RSSI level vs. node orientation in a back-to-back measurement setup.

Figure 20 displays the results of the measurements conducted using the back-to-back measurement setup. Distance between nodes is 1 m. Similar experimental setups are documented in [1,23,24]. If the basic sensor and the mobile sensor are placed back to back the RSSI diagram does not change much as the form (it is still directional), but the RSSI level is lower. The signal levels indicated by the RSSI, at different angular position values can be read from each diagram.

**Figure 21:**
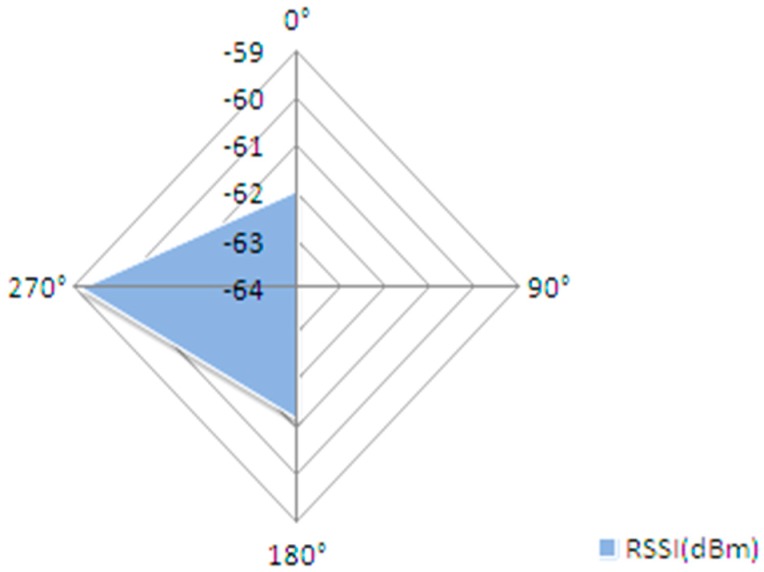
RSSI level vs. node orientation in a back-to-back measurement setup.

Figure 21 displays the results of the measurements conducted in [1], redrawn in the same format like Figure 20 for easier comparison of results. In [1] the measurements were only conducted on 4 angles. 

The distance between nodes is 5 feet and the RSSI values represent the average of 50 measurements in each direction.

For statistical analysis, using the setup in Figure 17, the RSSI factor measurements were repeated in a new indoor environment under different conditions. The test has been repeated 30 times, for each angular position, and the results are found in Table 3—Results of RSSI measurements—repeated every 30 times.

**Figure 22:**
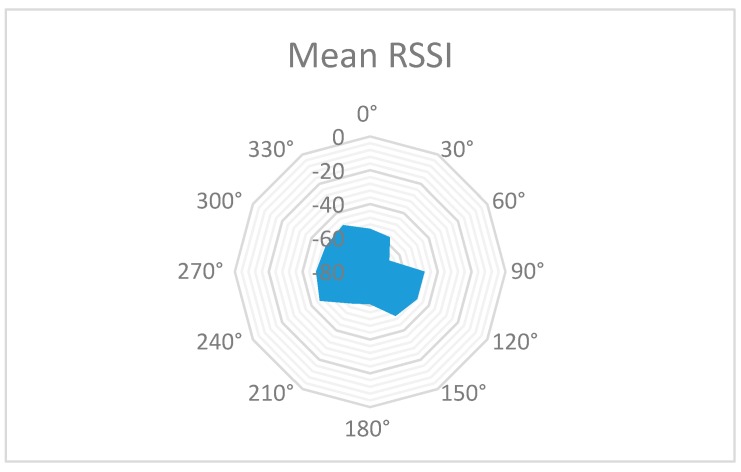
Statistical analysis. Mean RSSI value of measurements.

**Figure 23:**
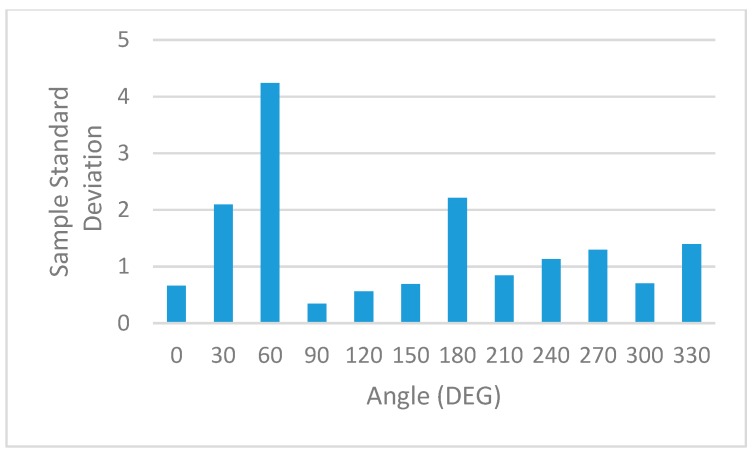
Statistical analysis. Sample standard deviation of RSSI measurements.

**Figure 24:**
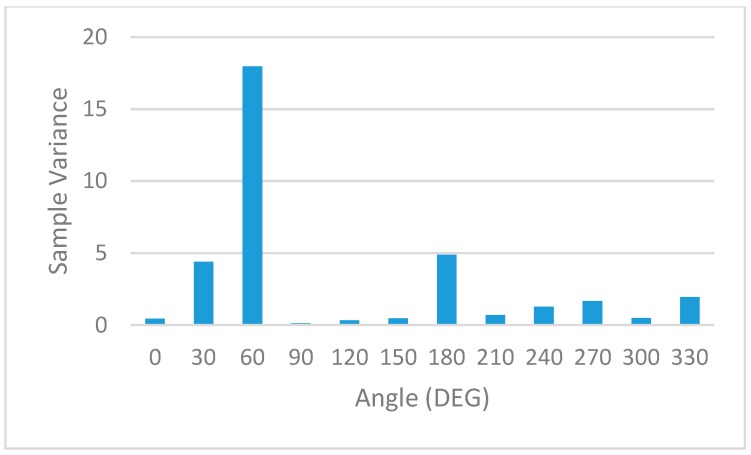
Statistical analysis. Sample variance of RSSI measurements.

**Figure 25:**
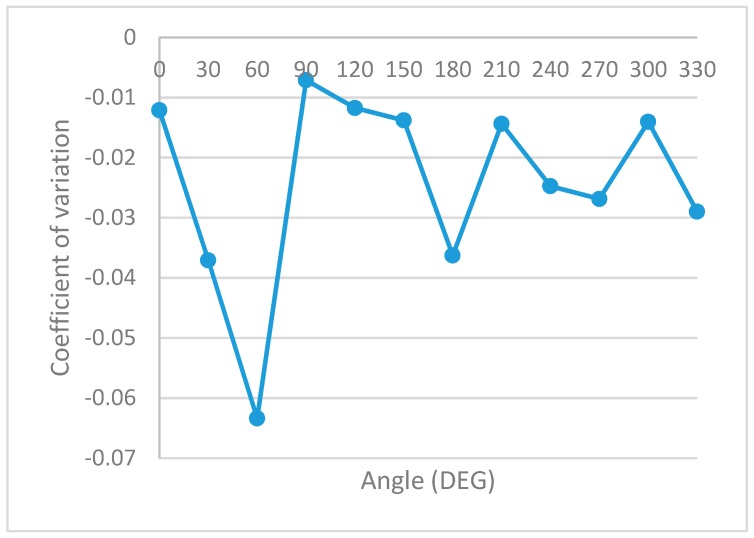
Statistical analysis. Coefficient of variation of RSSI measurements.

The statistical processing of this information is shown in Table 4 and graphically represented: Mean RSSI—Figure 22, Sample Standard Deviation—Figure 23, Coefficient of variation—Figure 24 and Sample Variance—Figure 25. (Statistical processing was performed using the WolframAlpha tools).

The Mean RSSI value, shown in Figure 22, was obtained by averaging 30 measurements in a face to face setup. Each measurement consists of eleven distinct measurements. The base station remained in a fixed position while the mobile node has been manually moved clockwise with 30 degrees every one minute whilst keeping the one meter radius constant. The RSSI reading has been logged 30 seconds after placing the node in the new position. This way any user perturbation on the measurement is avoided. 

In order to rule out interferences caused by environmental factors that might change over time, full sets of measurements were repeated periodically. A full set consists of eleven measurements that cover the full 360 degrees around the base station. The sample standard variation representation in Figure 23 and the corresponding sample variance in Figure 24 show a minimal spread in the distribution of RSSI measurements for each angle.

Both accuracy and reliability of the measurements during the experiments are validated using the coefficient of variation (CV) pictured in Figure 25. Given the low dispersion during these measurements, further studies on RSSI geometrical orientation in the same environment could be repeated using a lower number of gathered samples with the same degree of confidence.

All these results prove the variation of the signal level indicated by RSSI for the same distance between sensors, but with different angular orientations.

#### 3.2.4. Indoor Vertical Orientation Measurements

**Figure 26:**
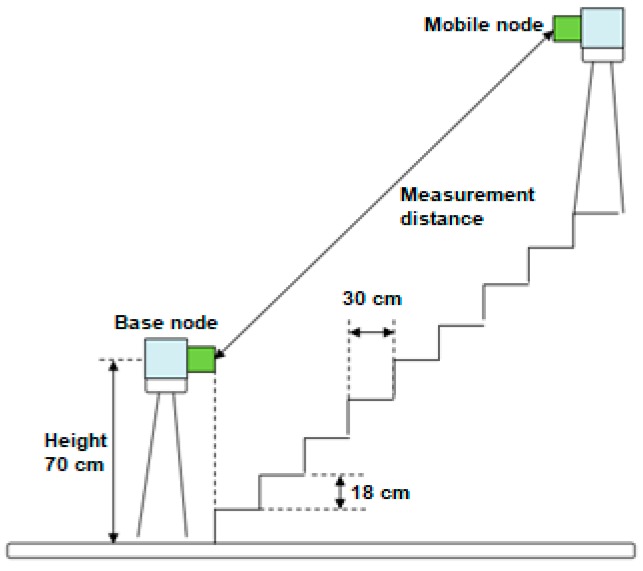
A vertical face-to-face measurement setup.

The goal of these measurements is to evaluate RSSI level between two nodes positioned at different heights and under a specific angle. In Figure 26 the measurement setup is illustrated. The base station is fixed and the remote station (mobile node) gradually moves upwards, one stair at a time. The stairs are located in a corridor of 1.3 m wide and 3 m long.

**Figure 27:**
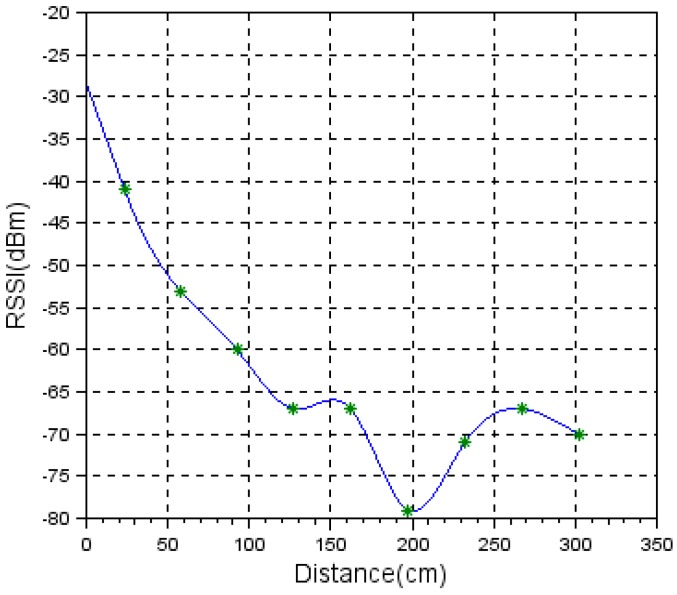
RSSI level vs. node orientation in a face-to-face vertical measurement setup.

In Figure 27, the measurement results obtained using the vertical face-to-face measurement setup from Figure 26 are shown.

Comparing the values at 1 m, 1.5 m, 2 m, 2.5 m, and 3 m shown in Figure 16 and Figure 27, we see big differences. The results clearly show that the directivity characteristics of the sensor’s radio module are not isotropic.

## 4. Discussion

The purpose of this paper is to highlight how far the results of scientific experiments can differ in wireless sensor networks by the orientation of a node to other nodes. As a result, there are implications for autonomy and localization. In an application where wireless node networks are used, where the node position is not known, the radio module is not indicated to have the directive feature.

The RSSI measurement system described in this paper, based on the proposed wired and wireless communication protocol proved its reliability and compatibility with wireless sensor networks. Through its design, the software system is scalable from the features and hardware resources point of view making it suitable for a wider range of wireless sensors with different hardware architecture and computing resources footprint.

In order to use the RSSI values as input for distance or location detection algorithms, the RSSI values can’t be used without intermediate processing steps to mitigate with the non-linearity of the measured values. The results of the measurements confirmed that the RSSI level varies with distance, geometrical orientation of the sensors and environment characteristics. 

Considering energy-efficient wireless sensor networks, it is expected that RSSI can be a solution for detecting the distance between nodes and afterwards, adjust the transmission power to a specific value corresponding to the measured distance. By reducing the transmission power, the current consumption of the transceiver is reduced and therefore the autonomy of the node is increased. Experimental results show that eZ430-RF2500T has a non-linear characteristic between current and transmission power as shown in Figure 10, Figure 11 and Figure 12, proving that it is not the best-suited hardware platform for this type of applications.

## 5. Conclusions

The paper shows that the RSSI system can be used to estimate the distance between the nodes of a wireless network. The precision of the measurements is however strongly affected by the orientation of the nodes in each location, besides the environmental factors. Carefully analyzing datasheet for a sensor node can relieve designers of problems that can only be found in field deployment.

The same signal level measured from the same two sensors does not mean that the distance between the two sensors is the same. Maybe if the sensors have the isotropic signal emission characteristic, the information will be improved. However, other environmental factors are involved that can influence the signal level indicated by RSSI measurements. We mention that we did not make measurements with such sensors nor in different environmental conditions.

The system designed and experimentally implemented for one type of sensor and one parameter can be easily adapted to the study of other wireless sensor nodes. The practical study of other parameters that provide data for node localization algorithms can be achieved by adapting the system presented in the paper. The system designed and experimentally implemented for one type of sensor can be easily adapted to the study of other wireless sensor nodes. The practical study of other parameters that provide data for node localization algorithms can be achieved by adapting the system presented in the paper.

The final results of such studies can lead us to more or less accurate estimates of the autonomy of a sensor or wireless sensor networks.

## Figures and Tables

**Table 1:** Notations and abbreviations.

No.	Abbreviation	Meaning
1	WSN	Wireless Sensor Networks; eZ430-RF2500T—Wireless Node from Texas Instruments Company
2	RSSI	Received Signal Strength Indicator
3	QoS	Quality of Service
4	AoA	Angle of Arrival
5	ToA	Time of Arrival
6	TDoA	Time Difference of Arrival
7	dB, dBm	Decibel (dB), Decibels with reference to one milliwatt (mW)
8	PL	Path of loss
9	f_c_	The central frequency
10	d_0_	The distance between nodes
11	Ni (i = 1, …, n)	Node number
12	Lj (j = 1, …, n)	Specific power level—Necessary to transmits from node Ni to node Nj
13	UART	Universal Asynchronous Receiver/Transmitter
14	PC	Personal Computer
15	RS232	Recommended Standard 232—Interface EIA (From Electronic Industries Alliance Standards)
16	MSP430F2274	Low power microcontroller (From Texas Instruments Company)
17	CC2500	Low power 2.4 Ghz transceiver (From Chipcon Company)
18	LED	Light-Emitting Diode
19	ISM	Industrial, Scientific and Medical radio bands
20	LQI	Link Quality Indication
21	TDMA	Time Division Multiple Access
22	ID	Identification Device (Number)
23	LSB	Least Significant Bit
24	RAM	Random Access Memory
25	SPI	Serial Peripheral Interface
26	D	Transmission Duration
27	C_Total_	Total Current Consumption—See Equation (3)
28	RX and TX	Transmit and Receive (From Electronic Industries Alliance (EIA) Standards)
29	VBAT	Fully Charged Batteries Level
30	Vbat	Discharged Batteries Level

**Table 2:** Control commands.

Command	Description
NODE_INFO	Get node information
PART_NUM	Get part number and HW version
GET_RSSI	Get RSSI
NET_SCAN	Network scan
FLASH	Flash operations
TX_LEVEL	Set TX power level
TX_STRESS	Send N data frames (stress test)

**Table 3:** RSSI measurements data.

#	0 DEG (dBm)	30 DEG (dBm)	60 DEG (dBm)	90 DEG (dBm)	120 DEG (dBm)	150 DEG (dBm)	180 DEG (dBm)	210 DEG (dBm)	240 DEG (dBm)	270 DEG (dBm)	300 DEG (dBm)	330 DEG (dBm)
1	−56	−53	−56	−47	−48	−49	−56	−59	−46	−44	−51	−51
2	−54	−53	−56	−47	−48	−49	−61	−58	−46	−46	−51	−51
3	−53	−53	−56	−47	−48	−49	−62	−59	−44	−47	−50	−49
4	−54	−53	−67	−47	−48	−49	−60	−60	−46	−47	−50	−49
5	−54	−53	−68	−48	−48	−52	−60	−58	−46	−47	−50	−49
6	−54	−53	−68	−48	−48	−50	−60	−59	−46	−47	−49	−49
7	−54	−53	−68	−48	−48	−50	−61	−59	−46	−47	−50	−49
8	−54	−56	−68	−48	−48	−49	−60	−59	−42	−47	−50	−49
9	−54	−58	−68	−48	−48	−49	−60	−59	−46	−47	−51	−49
10	−54	−58	−68	−48	−48	−49	−60	−58	−46	−47	−50	−50
11	−55	−58	−68	−48	−48	−50	−60	−58	−44	−47	−50	−49
12	−55	−58	−68	−48	−48	−51	−60	−59	−46	−47	−50	−49
13	−55	−58	−63	−48	−48	−49	−59	−59	−46	−47	−49	−49
14	−55	−58	−65	−48	−48	−49	−60	−59	−46	−48	−50	−49
15	−55	−58	−65	−48	−48	−50	−60	−59	−46	−49	−50	−50
16	−55	−58	−65	−48	−48	−50	−60	−59	−46	−49	−50	−50
17	−55	−58	−65	−48	−48	−50	−60	−58	−46	−49	−50	−50
18	−55	−58	−65	−48	−48	−50	−60	−56	−44	−50	−50	−53
19	−55	−58	−65	−48	−48	−50	−68	−56	−46	−49	−50	−48
20	−56	−58	−65	−48	−48	−50	−60	−58	−46	−50	−51	−47
21	−55	−58	−65	−48	−48	−50	−60	−58	−46	−50	−51	−48
22	−55	−58	−71	−48	−48	−50	−60	−58	−44	−49	−49	−47
23	−55	−58	−72	−48	−48	−50	−60	−58	−46	−49	−49	−48
24	−55	−58	−72	−48	−48	−50	−63	−58	−47	−49	−49	−47
25	−55	−58	−72	−48	−48	−50	−63	−58	−46	−49	−49	−48
26	−55	−59	−72	−48	−48	−50	−60	−59	−47	−49	−49	−47
27	−55	−56	−72	−48	−48	−50	−63	−59	−46	−49	−49	−47
28	−55	−56	−72	−48	−48	−50	−62	−58	−47	−49	−49	−47
29	−54	−56	−72	−48	−48	−50	−67	−58	−47	−48	−50	−47
30	−54	−56	−68	−48	−48	−51	−60	−58	−44	−48	−49	−47

**Table 4:** Statistical analysis of RSSI measurements.

Angle (DEG)	Mean (RSSI)	Sample Std. Deviation	Sample Variance	Coefficient of Variation	Confidence Interval (95%)
0	−54.65	0.6607	0.4366	−0.01209	−54.878 to −54.413
30	−56.52	2.096	4.391	−0.03708	−57.254 to −55.778
60	−66.86	4.237	17.95	−0.06337	−68.261 to −65.454
90	−47.78	0.3408	0.1161	−0.007119	−47.9909 to −47.7510
120	−47.77	0.5603	0.314	−0.01173	−47.971 to −47.577
150	−49.84	0.6878	0.4731	−0.0138	−50.081 to −49.597
180	−60.84	2.208	4.873	−0.03628	−61.616 to −60.062
210	−58.35	0.8386	0.7032	−0.01437	−58.650 to −58.060
240	−45.71	1.131	1.28	−0.02475	−46.108 to −45.311
270	−48.03	1.291	1.666	−0.02687	−48.463 to −47.596
300	−49.76	0.6989	0.4884	−0.01404	−50.000 to −49.530
330	−48.12	1.394	1.943	−0.02897	−48.513 to −47.732
